# Pulmonary Hypertension Detection Non-Invasively at Point-of-Care Using a Machine-Learned Algorithm

**DOI:** 10.3390/diagnostics14090897

**Published:** 2024-04-25

**Authors:** Navid Nemati, Timothy Burton, Farhad Fathieh, Horace R. Gillins, Ian Shadforth, Shyam Ramchandani, Charles R. Bridges

**Affiliations:** 1 Analytics for Life, Toronto, ON M5X 1C9, Canada; navid.nemati@analytics4life.com (N.N.); farhad.fathieh@analytics4life.com (F.F.); 2 Analytics for Life, Bethesda, MD 20814, USA; horace.gillins@analytics4life.com (H.R.G.); ian.shadforth@analytics4life.com (I.S.); charles.bridges@analytics4life.com (C.R.B.)

**Keywords:** artificial intelligence, digital health, front line, pulmonary hypertension, point-of-care

## Abstract

Artificial intelligence, particularly machine learning, has gained prominence in medical research due to its potential to develop non-invasive diagnostics. Pulmonary hypertension presents a diagnostic challenge due to its heterogeneous nature and similarity in symptoms to other cardiovascular conditions. Here, we describe the development of a supervised machine learning model using non-invasive signals (orthogonal voltage gradient and photoplethysmographic) and a hand-crafted library of 3298 features. The developed model achieved a sensitivity of 87% and a specificity of 83%, with an overall Area Under the Receiver Operator Characteristic Curve (AUC-ROC) of 0.93. Subgroup analysis showed consistent performance across genders, age groups and classes of PH. Feature importance analysis revealed changes in metrics that measure conduction, repolarization and respiration as significant contributors to the model. The model demonstrates promising performance in identifying pulmonary hypertension, offering potential for early detection and intervention when embedded in a point-of-care diagnostic system.

## 1. Introduction

Since the advent of modern computational technologies and the increasing accumulation of healthcare data, artificial intelligence has evolved into an active area of research within the medical domain. Deep learning algorithms are a popular choice, in part due to their ability to discover features from unprocessed data, eliminating the need for domain expertise. However, deep learning algorithms require large datasets with tens of thousands to hundreds of thousands of examples to perform well, and as a result, practitioners routinely utilize tangentially related data sources to supply sufficient data (consequently biasing the models). Further, the interpretability of the prediction mechanism of deep learning algorithms, including the nature of the learned features and their importance, is an area of active research, and cannot yet be performed consistently in a trustworthy manner.

Conversely, classical supervised machine learning uses a known set of input features instead of raw data, which reduces data needs by multiple magnitudes to alleviate deep learning data constraints in complex medical applications where the quantity of ground-truth data is limiting. The final models generated are transparent and allow understanding of the physiological mechanisms underpinning the output. However, developing significant and relevant features requires a thorough understanding of signal processing, mathematics and medicine.

Pulmonary hypertension (PH) is a group of heterogeneous disorders characterized by a mean pulmonary arterial pressure (mPAP) of ≥25 mmHg based on the 2015 ESC/ERS Guidelines [1] and ≥21 mmHg based on the 2022 ESC/ERS Guidelines [2], measured using invasive right heart catheterization (iRHC) [3]. PH is most prevalent in those with left heart failure (systolic or diastolic), a group in which the PH prevalence estimates are between 25 and 83% [4,5]. The prevalence of PH is strongly and independently correlated with age [6]. It has also been suggested that elevated pulmonary pressure is itself a cardiovascular risk factor due to its independent association with increased mortality [6]. PH can be divided into subgroups based on pulmonary capillary wedge pressure and pulmonary vascular resistance, as shown in Table 1, which also identifies the corresponding World Health Organization (WHO) groups [7].

PH is ubiquitous, affecting an estimated 1% of the world population and up to 10% of people over 65 years of age, as well as 50% of patients with heart failure [8]. The vast majority of people with PH (80%) live in areas with limited access to appropriate medical care [9]. PH is a life-threatening condition with significant morbidity and mortality regardless of etiology or group classification [3]. There is potential to alter the course of this disease, improve survival and increase health equity if PH is detected early enough, which requires readily available testing methods to permit necessary interventions and therapies. Importantly, PH patients present with symptoms similar to those of other cardiovascular disease states (i.e., coronary artery disease and left-sided heart failure), further increasing the complexity of its recognition and ultimately its diagnosis. iRHC serves as the gold standard for diagnosing PH. Furthermore, patients with a specific form of PH, pulmonary arterial hypertension (PAH), which affects younger females in particular, are frequently diagnosed years after symptom onset, at a point when the pathophysiologic changes have become irreversible. There is a clear need for novel point-of-care diagnostics that identify patients with PH earlier in the clinical pathway. Specifically, point-of-care testing is performed where the clinician is assessing the patient, not requiring referral to off-site testing services or higher levels of care (secondary/tertiary care), increasing the accessibility of the test.

Transthoracic Echocardiography (TTE) is a routinely performed point-of-care test that may provide some information on PH status. Janda et al., in their meta-analysis comprising 29 studies and a total of 1995 patients, compared the efficacy of TTE to iRHC in diagnosing PH. Their findings revealed that in 41% of cases, the tricuspid regurgitant (TR) jet, necessary for systolic pulmonary arterial pressure evaluation, was unmeasurable [10]. A similar challenge was observed by Lam et al., where TR jets were analyzable in only 69% of subjects [6]. Notably, most cases with unmeasurable TR jets stemmed from studies predominantly involving chronic obstructive pulmonary disease (COPD) patients. These results underscore the limitations of TTE in accurately assessing elevated PAP across diverse populations. Sensitivity and specificity were dependent on disease state and demonstrated a wide range of performance; from 0.58 to 0.97 for sensitivity and 0.46 to 1 for specificity [10]. When the various results assessed in the review were combined, the sensitivity was found to be 0.83 (0.73 to 0.90), and the specificity was found to be 0.72 (0.53 to 0.85) [10]. Janda found that a TTE assessment yielded a result in only 59% of the subjects tested; considering the entire population, versus solely that part for which a TTE result may be obtained, the sensitivity may be more correctly stated as 0.49 (0.83 × 0.59), with specificity of 0.42 (0.72 × 0.59).

While TTE remains widely available in hospitals and cardiology clinics, its diagnostic accuracy hinges on skilled parameter measurement, requiring expert operators. However, the inability to assess mPAP in a significant proportion of cases, often only discovered post-test, results in wasted time and resources and delayed treatment. In contrast, we believe that it is possible to develop an algorithm to assess for elevated mPAP at point-of-care with high performance, without reliance on expert operators, and in patients currently left behind by TTE due to unmeasurable TR.

Although a few rule-based models have been described for detecting PH, their reliability is still questionable. For example, a recent study compared different methodologies using a rule-based and machine learning (ML) models for identifying PH, finding that all the ML models outperformed the rule-based models [11]. However, the proposed ML models rely heavily on patient age, medical history (e.g., heart failure, primary PH, valvular heart disease and cardiomyopathy) and outcomes of other non-invasive tests (e.g., electrocardiography and echocardiography). However, the applicability of such models can face limitations when such information is unavailable.

Thus, herein, we sought to employ machine learning to develop a high-performance model for the detection of PH in symptomatic patients without the use of patient metadata or medical history. Such a model can be employed in a system to assess PH at point-of-care, without the need for expert TTE operators, and reliance on TTE measures such as TR jet velocity. The development methodology parallels that used to successfully develop a model to assess for coronary artery disease (CAD) [12].

## 2. Materials and Methods

### 2.1. Clinical Studies & Population

The subjects used in the present work were drawn from the CADLAD (NCT02784197), IDENTIFY (NCT03864081) and IDENTIFY-PH (NCT04031989) prospective studies, as well as the RADPH retrospective study, all of which were approved by the Western Institutional Review Board. Informed consent was obtained from all subjects. CADLAD enrolled subjects prior to invasive coronary angiography (ICA) by left heart catheterization, and iRHC was also performed in a subset of subjects. The IDENTIFY study both continues and extends CADLAD; IDENTIFY Group 2 is identical to CADLAD, while IDENTIFY Group 4 enrolled subjects with new-onset cardiovascular symptoms referred by their physician for Computed Coronary Tomography Angiography (CCTA) for assessment of CAD. IDENTIFY Group 3 enrolled subjects with new-onset cardiovascular symptoms referred by their physician for Single-Photon Emission Computed Tomography Myocardial Perfusion Imaging (SPECT MPI) for assessment of CAD. IDENTIFY-PH enrolled subjects with new-onset cardiovascular symptoms referred for iRHC. RADPH enrolled subjects who had previously undergone iRHC (within 18 months of screening for the study), where that iRHC showed an mPAP of at least 30 mmHg. See inclusion/exclusion criteria for all studies in Supplement Section S1.

The CorVista Capture device (Analytics for Life; Toronto, ON, Canada & Bethesda, MD, USA) [13] non-invasively acquired orthogonal voltage gradient (OVG) and photoplethysmogram (PPG) signals simultaneously from each subject at rest prior to the reference test (CCTA and MPI-SPECT in IDENTIFY or iRHC in IDENTIFY-PH and CADLAD) or after the reference test (iRHC in RADPH). Subjects in IDENTIFY Groups 3 and 4 must have had a TTE within 90 days of signal collection that showed a low probability of PH using the ESC/ERS guidelines [2], in addition to being negative for diastolic dysfunction [14].

Training and internal validation refers to the process of iteratively training and generating naïve predictions within the cross-validation procedure for performance evaluation. Table 2 shows the contribution of subjects from each study. IDENTIFY-PH contributed N = 252 PH+ subjects in total, composed of N = 120 females and N = 132 males. PH- subjects were sourced from IDENTIFY Group 3, composed of N = 43 males (and no females), and IDENTIFY Group 4 (N = 161), composed of N = 106 females and N = 55 males. IDENTIFY-PH and IDENTIFY Group 4 were only used for the internal validation process because of the enhanced confidence in the absence of CAD granted by CCTA over SPECT. IDENTIFY Group 3 was used in training only to compensate for the shortfall of male subjects in IDENTIFY Group 4.

Given that the point-of-care system in which we planned to embed the resultant algorithm to assess for elevated mPAP is intended to be used on any symptomatic patient indicated for TTE assessment of PH, and TTE can reliably detect the absence (but not presence) of mPAP elevation, the subjects from IDENTIFY-PH provided the elevated cohort using the gold standard of iRHC, while the TTE subjects negative for PH provided the non-diseased cohort. In combination, we refer to these two cohorts as the Intended Use Population.

Equal treatment of both genders was of critical importance in the development of the PH Algorithm, and as discussed, IDENTIFY Group 3 was required to supplement the relative lack of males in IDENTIY Group 4 (Table 2). Given the use of IDENTIFY Group 3, the dataset was approximately balanced by gender and disease, and therefore there was no need for any measures to impose balance (otherwise, sample weighting or other similar approaches would have been explored). Finally, note that the description of the validation (in training and internal validation) is intended to convey that all performances derived from that data are estimates only, given that the gold-standard methodology for validating a ML algorithm is a large, blinded dataset that is assessed only once—which is under review in a manuscript describing the clinical validation of this model.

### 2.2. Overview of Model Development Process

**Figure 1:**
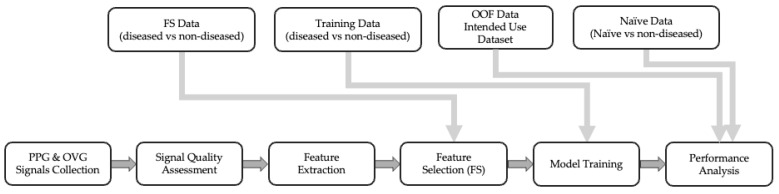
Step-by-step process of model development with the data flow.

The PH algorithm is the series of processing steps to take in a signal from the CorVista Capture device and return a prediction reflective of PH status (i.e., PH Score). The development process began with assessment of the quality of captured signal, then feature extraction from OVG and PPG signals [15], followed by univariate feature selection to identify discriminative features. Statistical tests were employed to retain only the significant features, reducing the dimensionality of the dataset. Subsequently, Elastic Net (EN) and Random Forest (RF) models were trained using the selected features, intended to capture both linear and non-linear relationships. An out-of-fold (OOF) prediction methodology ensured comprehensive evaluation across the dataset while maintaining validation integrity. Gender balance was carefully considered throughout the process. Figure 1 provides a schematic of the pipeline used in our study, and detailed descriptions of each step are provided in the following sections.

### 2.3. Signal Collection, Quality Assessment and Feature Extraction

Two sources of time-series data were simultaneously acquired (within 1 millisecond) from each subject: (i) OVG signals and (ii) PPG signals (Red and IR). These signals were collected at a sampling rate of 8 kHz using a specialized instrument (CorVista Capture, both hardware and firmware) [13]. OVG signal quality was assessed for possible environmental interference (i.e., 60 Hz powerline and high-frequency noise, ≥170 Hz). The quality of the PPG signal was also checked for possible artifacts, i.e., jumps, saturation and clipping, as described in [13]. Signals with low quality were excluded from further analysis, and upon passing the quality assessments, features were extracted from the signals. Herein, OVG and PPG signals were analyzed in their different representations including time-domain, frequency, time–frequency and phase space. Several techniques have been employed for feature engineering, such as spectral, scalogram, time-series, dynamical and topological analysis. Features have previously demonstrated utility in the assessment of CAD [12,15] and elevated left ventricular end diastolic pressure [15,16]. Detailed description of the features’ calculation and their reported utility can be found in Supplement Section S2.

### 2.4. Dimensionality Reduction (Feature Selection)

Given the large feature library (3298 features), particularly as compared to the number of subjects, a dimensionality reduction step to reduce the number of features was undertaken using univariate feature selection. Features were assessed for statistical ability to separate diseased subjects from non-diseased subjects. The statistical testing was performed on an N = 161 dataset from RADPH, CADLAD and IDENTIFY Group 2, composed of a roughly equal division into diseased and non-diseased, as described in Table 3. The N = 83 diseased subjects had mPAP ≥ 21 mmHg across all three study groups. The N = 78 non-diseased subjects had mPAP ≤ 16 mmHg, chosen to be somewhat close to the elevated group from CADLAD and IDENTIFY Group 2. The negative subgroup used for the dimensionality reduction was selected from the iRHC-negative population and did not include any of the negative subgroups from TTE used in the training. The feature selection data was chosen to reduce the effect size between the negative and positive iRHC, which would result in the selection of the features with the most predictive power and reduce type I error. As mentioned, care was taken at this stage (which carries through the process) to ensure that the genders were treated evenly; in this case, the number of males and females in each of the diseased and non-diseased groups were within one of each other. It should be noted that none of the subjects utilized in feature selection were incorporated into the training process, which ensures the integrity of feature selection by preventing any potential bias leakage from the feature selection dataset (dimensionality reduction) to the training dataset.

Univariate tests were used to determine whether the feature significantly separated the two cohorts. While there are many methods for dimensionality reduction available in the literature, there is not a specific one applicable to all model types [17]. Herein, the proposed univariate feature selection was used due to its computational efficiency, straightforward interpretability and ability to effectively reduce the number of features to the most predictive. Three metrics were used for feature selection: (I) *t*-test (to detect a difference in the means of the distributions); (II) ROC-AUC (treating the feature as a predictor of the disease state); and (III) mutual information (to detect differences in the shapes of the distributions). To select features using *t*-test, a threshold for the *p*-value was established at 0.025 (half of the conventional threshold of 0.05). To select features used ROC-AUC, bootstrap sampling was used to calculate the 95% confidence interval, of which the lower bound needed to be greater than 0.505 or the upper confidence bound needed to be less than 0.495. To select features using mutual information, bootstrap sampling was used to calculate the 95% confidence interval, the lower bound of which needed to be greater than 1.4. Finally, to remove features with small means and small variations (where minor changes due to computational precision could lead to significant deviations), the mean and standard deviation of the feature needed to be greater than 0.001.

### 2.5. Modeling

The primary goal of machine learning is to ensure that performance generalizes to unseen datasets, and stacked ensembling is a valuable tool to achieve this aim. Two model types were selected for inclusion in the PH Algorithm: EN and RF. The use of stacked ensembling, which, in this case, is the averaging of the predictions from RF and EN, increases generalizability by reducing reliance on either of the single model types. The selection of EN and RF as the component models of the PH Algorithm was intended to capture linear and non-linear relationships, respectively, between the features and the PH status. The usage of EN is particularly well suited for datasets with a large number of features compared to the number of samples, commonly referred to as high-dimensional data [18]. Specifically, EN combines the strengths of both Lasso and Ridge regression, allowing it to effectively handle multicollinearity and select relevant features even when the number of predictors exceeds the number of observations. Similarly, RF has an inherent regularization to reduce the risk of overfitting due to the use of a large number of trees and bootstrapping [19].

Note that there is no way to determine a priori which machine learning algorithm is best suited for any particular problem, though reasoning can be applied to reduce the suite of options (i.e., to models known to work well with small datasets). Therefore, we applied several classifiers/regressors, which are widely used for the assessment of cardiovascular diseases and cover a wide range of linear and non-linear methods, for the development of PH models; however, we found that EN and RF outperformed the other models.

EN is a regularized linear regression that combines weight (w) regularization using both $l_{1}$ (${\left\|w\right\|}_{1}=\sum_{i}w_{i}$) and $l_{2}$ (${\left\|w\right\|}_{2}^{2}=\sum_{i}w_{i}^{2}$) penalties [20].

RF is an ensemble algorithm composed of underlying tree models. Each tree optimizes the mean squared error loss function ($\frac{1}{n}\sum_{i=1}^{n}{\left\{y-ŷ\right\}}^{2})$ by selecting features upon which to split the dataset until a terminal leaf node is reached, containing prediction for the remaining subset of data. A large collection of trees are trained on differing subsets of the subjects and the predictions from the trees are averaged to result in the overall RF prediction.

### 2.6. Performance Analysis

To enable robust characterization of the PH model, an out-of-fold (OOF) prediction methodology was developed to enable generation of predictions on the entire Intended Use Dataset (IDENTIFY-PH and IDENTIFY Group 4); this is a critical functionality to allow use of the entire Intended Use Dataset in training, while still providing for analysis of the Intended Use Dataset as an internal validation set for ROC curve generation (including cut-point selection) and subgroup analysis (most importantly, by gender). The alternative to OOF prediction is the use of a static training set (within which cross-validation could still be performed) and a static internal validation set; the disadvantage of this strategy is the inherent limitation of the usage of each of those datasets. Specifically, the internal validation data cannot be used for training, impacting the ability of the model to generalize, and the training data cannot be used for assessment of the model (ROC curve, subgroups, etc.), limiting confidence in that assessment based on limited dataset size.

**Figure 2:**
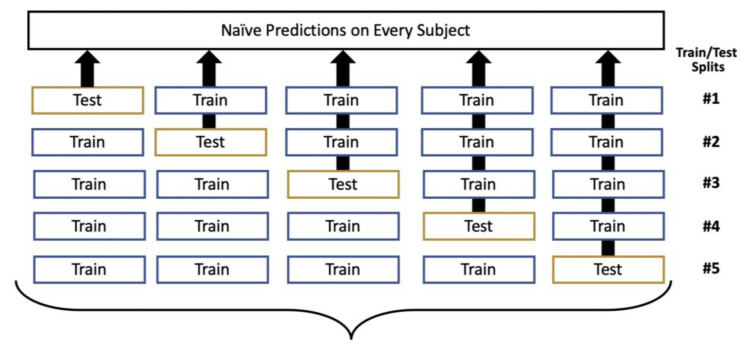
Out-of-fold (OOF) prediction generation, with this process repeated over 100 iterations.

OOF prediction is built upon cross-validation, using the same fivefold stratified division [21]. However, it was extended as shown in Figure 2; given a model that was trained on four folds with the fifth withheld for testing, the predictions were stored from that fifth fold. As the folds were iteratively reserved for testing, the result was complete coverage of the entire dataset with respect to naïve prediction generation. As in cross-validation, this process was repeated over 100 iterations to vary how the data was divided into the five folds.

It should be noted that the hyperparameters and model configurations were locked using the cross-validation paradigm, and during OOF retraining, loss function optimization was not performed. This was an important control to ensure that there was no bias transfer from the previous training step that could potentially increase the risk of overfitting the training set. The output of the OOF strategy was 500 models; when evaluating the Intended Use Dataset, the median prediction was taken across the 100 models for which each subject was unseen. Further, when evaluating any other dataset, the median prediction across all 500 models was used. The use of this large number of models, each trained on a different subset of the data, is known as a bagged ensemble [22].

## 3. Results

### 3.1. Selected Features

**Figure 3:**
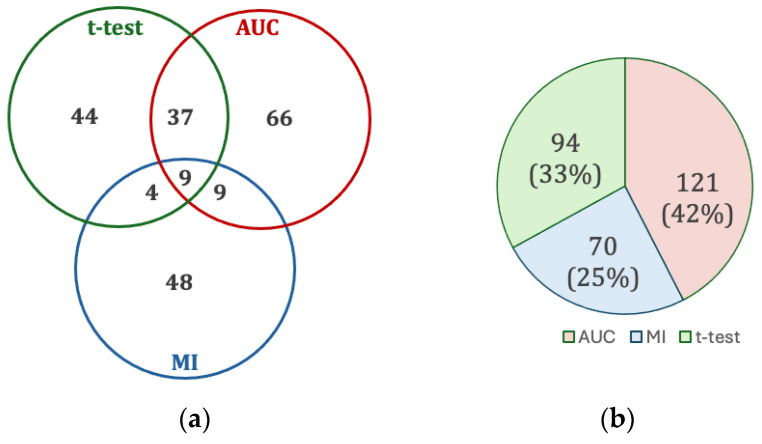
(**a**) Intersection of univariate test feature selection. (**b**) Contribution of each univariate test to the set of all selected features.

Figure 3 shows the results of the feature selection, which yielded 217 features from a library of 3298 hand-crafted features. The most common scenario for selection was by AUC alone with 66 features (30%). Only 59 (27%) features were selected by two or more tests, reflecting the necessity of applying all these tests, as that majority of features (158, 73%) originated from only one test (i.e., chosen by only one of the *t*-test, AUC, or MI).

### 3.2. Relationship between EN and RF

**Figure 4:**
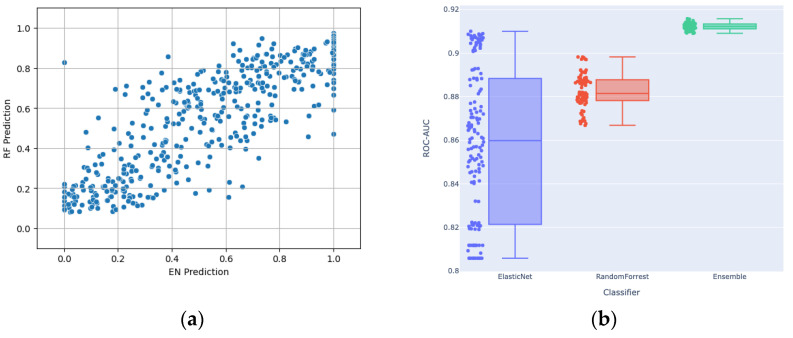
(**a**) Scatter plot showing the relationship between RF and EN predictions; (**b**) Distribution of the ROC-AUCs of EN, RF and their stacking ensemble (with outputs averaged) across the assessed hyperparameters.

As described, the modeling approach employed an ensemble strategy, combining the strengths of EN and RF algorithms. EN, with its regularization technique, excels at capturing linear relationships within the data, emphasizing key features that contribute to a linear model. On the other hand, the RF algorithm is adept at capturing complex non-linear patterns and interactions among features, providing a robust framework for capturing intricate relationships that may not be evident in a linear context. By leveraging the distinctive advantages of both models, the ensemble seeks to harness the complementary nature of EN’s focus on linear relationships and RF’s ability to capture diverse, non-linear patterns, resulting in a comprehensive and accurate predictive model. Figure 4a shows a scatter plot of the EN and RF components of the ensemble individually, which exhibited Pearson and Spearman correlations of 0.85. Figure 4b shows that the ensembling of EN and RF reached higher performance than each of them individually. Each dot represents a different set of selected hyperparameters, which were then used for ensembling. In assessing the main contributing features for both EN and RF models, there was only one common feature among the top 10 contributed features between the two model types, which is another demonstration of the distinct mechanisms by which the outputs from each were generated.

### 3.3. Performance

**Figure 5:**
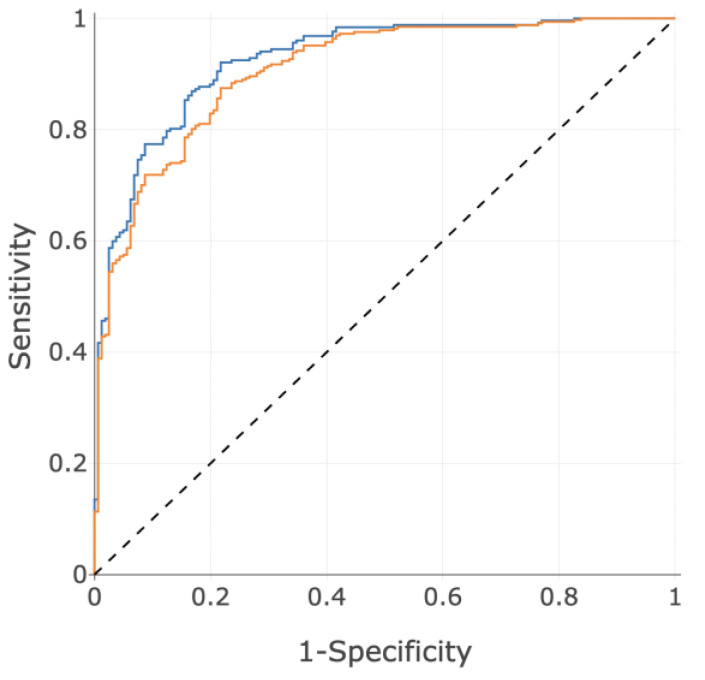
ROC curves using the PH score for the primary disease population defined by 25 mmHg (blue, AUC = 0.93) and the secondary disease population defined by 21 mmHg (orange, AUC = 0.91).

The resultant ROC curves are shown below in Figure 5, and subgroup performances are shown in Table 4. As discussed, the 2015 guidelines [1] use a threshold of 25 mmHg, which has since been updated to 21 mmHg in the 2022 guidelines [2]; however, a significant corpus of literature, including drug safety and efficacy reporting, has been created with the 2015 threshold of 25 mmHg, and therefore, that definition was adopted as the primary disease population for the present work. Figure 5 shows the ROC curves of OOF predictions (blue) together when naïve predictions of the additional 75 subjects from IDENTIFY-PH with 21 mmHg ≤ mPAP ≤ 24 mmHg added (orange), to demonstrate the performance on the 21 mmHg definition from the 2022 guidelines. Table 5 shows the performance, including subgroups, for 21 mmHg using 2022 guidelines.

### 3.4. Feature Importance

Understanding model behavior presents a significant challenge in healthcare and is not yet frequently performed successfully. Here, to emphasize the importance of eXplainable Artificial Intelligence (XAI), we conducted a feature importance analysis [23]. To further extend the interpretability of the current model, the model features were categorized based on their possible underlying physiology. Further information is available in Supplement Section S2.

**Figure 6:**
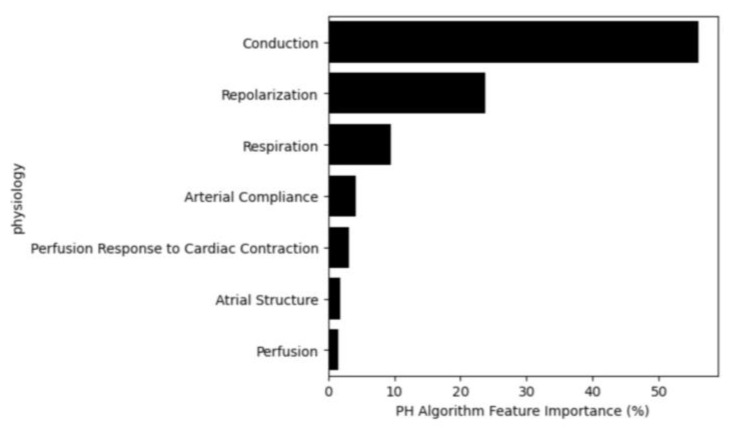
Feature importance based on their physiological category.

Figure 6 illustrates the feature importance for the PH Algorithm by physiological category. Conduction is the most contributive category, encapsulating features calculating characteristics of myocardial conduction pathway and variations in that pathway. Repolarization is the next most influential category, quantifying the recovery of the myocardium, including power distribution, heterogeneity, timing, morphology and variation [24]. Respiration features estimate the respiration waveform and evaluate characteristics of that estimation, using the PPG and OVG signals. Arterial compliance features employ the first or second derivative of the PPG (i.e., velocity plethysmogram and acceleration plethysmogram), both of which are known to embed characteristics of arterial compliance [25]. ‘Perfusion response to cardiac contraction’ features characterize the interplay between the OVG signal and the PPG signal, therefore embedding the perfusion response to cardiac pulsation [15]. Atrial structure features capture heterogeneity in atrial composition, including atrial enlargement [26]. Finally, perfusion features capture morphology of the PPG waveform, and the relationship between the infrared and red signals [27]. These findings are not unexpected from a clinical perspective as PH can often be the result of diastolic dysfunction, presenting as modified conduction and abnormal repolarization. Further, changes in respiration are a logical sequelae of PH.

## 4. Discussion

A PH Algorithm was developed on a clinically relevant population, designed to perform equally on both men and women with a diagnostic profile. Initial performance using OOF predictions demonstrates that these design goals were met, with an overall performance of ROC-AUC of 0.93, with a sensitivity of 87% and specificity of 83%. It must be noted that there are no non-invasive methods that achieve similar performance. The test described here, CorVista, requires no radiation exposure, no stress of any kind and no contrast agents, and it can be performed in any setting, including rural (the only requirement being an internet connection) with immediate results, a true point-of-care test. Critically, the test addresses the disparity in healthcare access for rural vs. urban populations, given the portability and ease of the test.

The results presented show robust overall performance across both males and females. Importantly, the algorithm performance is robust as a function of age, an important characteristic since some subtypes of PH have differing age and gender biases. The importance of this observation is enhanced considering that Table 2 shows significant differences in age and BMI across the negative and positive training groups; however, Table 4 and Table 5 show no statistically significant difference in the model performance across the age and BMI subgroups. Therefore, the imbalance of BMI and age in training have not been used by the model for the detection of PH, i.e., no significant confounding effect. PAH is more common in younger females, and other types of PH (e.g., isolated post-capillary PH) are more common in patients above the age of 65. Given these demographic variations, it is thus salient that the AUC, sensitivity and specificity of the algorithm is roughly equivalent for all subgroups of PH, pre-capillary, combined pre- and post-capillary and isolated post capillary PH. Further, this is also important given that there are now approved treatments for Group 1 (PAH, pre-capillary), Group 3 (pre-capillary) and that the drug sotatercept was found to be a highly effective treatment for the treatment of PAH [28]. In addition, the SGLT2i class of drugs was recommended for the treatment of HFpEF, the cause of most cases of Group 2 PH, in the 2022 ACC HF guidelines [29]. The availability of these new highly effective treatments for PH significantly augments the population health benefits of earlier diagnosis of all types of PH.

The data used in the present work is a manageable clinical dataset with respect to size, representing significant effort to enroll, yet still presents challenges for deep learning approaches. In contrast, to address the dataset size, we manually engineered or “hand-crafted” a large feature library and performed dimensionality reduction of the feature space using feature selection, followed by classical machine learning using a stacked ensemble of EN and RF.

A key advantage of classical machine learning, such as EN and RF used here, is ease of model interpretation, whereas that process is much more complex in deep learning. The feature importance analysis provides insight into the prediction mechanism of the PH Algorithm. This algorithm puts high importance on differences in myocardial conduction and the characteristics of repolarization. Intuitively these changes make sense as a hallmark of sustained pressure increase leading to myocardial remodeling. However, the nature of the changes is heterogenous and therefore a single measure of the myocardium or a specific element of conduction are not able to evaluate disease with any efficacy; however, if several features change in several patients who all have PH, then a machine learning algorithm can assemble the relationships between the features and the disease.

## 5. Conclusions

In conclusion, our study demonstrates that utility of machine learning for the detection of pulmonary hypertension in symptomatic patients with AUC-ROC of 0.93, sensitivity of 87% and specificity 83%. Importantly, subgroup analysis revealed consistent performance across genders, ages and classes of PH, underscoring the model’s generalizability and applicability in diverse patient populations.

When implemented into an integrated system with OVG and PPG sensors, such as the CorVista System used in this study, the proposed model can serve as a non-invasive point-of-care diagnostic test. By leveraging machine learning algorithms, we can streamline diagnostic processes and ultimately improve patient outcomes. The CorVista System with the PH Algorithm described in this manuscript was validated on a large, independent, blinded dataset and subsequently received FDA 510(k) clearance through the device breakthrough program (April 2024).

Our study represents a significant step towards harnessing the power of artificial intelligence for enhancing medical diagnostics and improving patient care in complex diseases such as pulmonary hypertension.

## Figures and Tables

**Table 1:** Definition of PH & subgroups per ECS/ERS guidelines (2015 & 2022), with WHO groups for each. PCWP = pulmonary capillary wedge pressure, PVR = pulmonary vascular resistance & WU = Woods units.

	2015 ESC/ERS Guidelines	2022 ESC/ERS Guidelines	WHO Groups
Pre-Capillary PH	mPAP ≥ 25 mmHg PCWP ≤ 15 mmHg PVR > 3 WU	mPAP ≥ 21 mmHg PCWP ≤ 15 mmHg PVR > 2 WU	1, 3, 4, 5
(Isolated) Post-Capillary PH	mPAP ≥ 25 mmHg PCWP > 15 mmHg PVR ≤ 3 WU	mPAP ≥ 21 mmHg PCWP > 15 mmHg PVR ≤ 2 WU	2, 5
Combined Pre- & Post-Capillary PH	mPAP ≥ 25 mmHg PCWP > 15 mmHg PVR > 3 WU	mPAP ≥ 21 mmHg PCWP > 15 mmHg PVR > 2 WU	2, 5

**Table 2:** Demographics and disease used in the training.

Characteristic	PH− IDENTIFY Groups 3 & 4	PH+ IDENTIFY PH	*p*-Value
**Number of Subjects**	204	252	
**Age**			
Mean ± Std	54.6 ± 12.2	64.7 ± 12.3	<0.05
Age ≥ 65	19.6% (40/204)	53.6% (135/252)	<0.05
Age < 65	80.4% (164/204)	46.4% (117/252)	
**Sex**			0.358
Male	48.0% (98/204)	52.4% (132/252)	0.397
Female	52.0% (106/204)	47.6% (120/252)	
**BMI**			<0.05
Mean ± Std	31.3 ± 6.7	33.5 ± 9.2	<0.05
BMI ≥ 30	53.0% (108/204)	58.7% (148/252)	0.217
BMI < 30	47.0% (96/204)	40.9% (103/252)	
PH Subgroups *			
Combined Pre- & Post-Capillary		51 (20.2%)	
Isolated Post-Capillary		65 (25.8%)	
Pre-Capillary		40 (15.9%)	

* Subgroups do not add up to 100% due to (1) inability to categorize subjects lacking pulmonary capillary wedge pressure and/or pulmonary vascular resistance measurement or (2) subjects belonging to the unclassified PH subgroup.

**Table 3:** Subjects used for dimensionality reduction.

Status	Females	Males	Total	Threshold
Positive	41	42	83	mPaP ≥ 21
Negative	39	39	78	mPaP ≤ 16

**Table 4:** Subgroup Performance on Intended Use Dataset (25 mmHg using 2015 guidelines).

Subgroup	Size	ROC-AUC (95% CI)	Sensitivity (95% CI)	Specificity (95% CI)
All Subjects	413	0.93 (0.91–0.95)	87% (84–90%)	83% (79–87%)
Sex				
Males	187	0.95 (0.92–0.98)	89% (86–93%)	87% (82–92%)
Females	226	0.90 (0.86–0.94)	85% (80–90%)	80% (75–85%)
Age				
≥65	162	0.89 (0.84–0.94)	84% (78–90%)	78% (72–84%)
<65	251	0.94 (0.91–0.97)	91% (87–95%)	84% (79–89%)
BMI				
≥30 *	233	0.90 (0.86–0.94)	82% (77–87%)	80% (75–85%)
<30 *	179	0.96 (0.93–0.99)	93% (89–97%)	86% (81–91%)
PH Groups				
Combined Pre- & Post-capillary **	51	0.94 (0.87–1.00)	86% (76–96%)	83% (73–93%)
(Isolated) Post-capillary **	65	0.92 (0.85–0.99)	88% (80–96%)	83% (74–92%)
Pre-capillary **	40	0.95 (0.88–1.00)	92% (84–100%)	83% (71–95%)

* One subject did not have BMI available. ** PH subgroup was adjudicated when PCWP and PVR were both available, and unclassified PH was excluded. Ns include positives only (negatives constant at N = 161).

**Table 5:** Subgroup Performance on Intended Use Dataset (21 mmHg using 2022 guidelines).

Subgroup	Size	ROC-AUC (95% CI)	Sensitivity (95% CI)	Specificity (95% CI)
All Subjects	488	0.91 (0.88–0.94)	80% (76–84%)	83% (80–86%)
Sex				
Males	225	0.94 (0.91–0.97)	83% (78–88%)	87% (83–91%)
Females	263	0.88 (0.84–0.92)	78% (73–83%)	80% (75–85%)
Age				
≥65	205	0.87 (0.82–0.92)	78% (72–84%)	78% (72–84%)
<65	283	0.92 (0.89–0.95)	83% (79–87%)	84% (80–88%)
BMI				
≥30 *	271	0.89 (0.85–0.93)	78% (73–83%)	80% (75–85%)
<30 *	216	0.93 (0.90–0.96)	84% (79–89%)	86% (81–91%)
PH Groups				
Combined Pre- & Post-capillary PH **	81	0.94 (0.89–0.99)	89% (82–96%)	83% (75–91%)
(Isolated) Post-capillary PH **	45	0.89 (0.80–0.98)	76% (64–88%)	83% (72–94%)
Pre-capillary PH **	74	0.94 (0.89–0.99)	88% (81–95%)	83% (74–92%)

* One subject did not have BMI available. ** PH subgroup was adjudicated when PCWP and PVR were both available, and unclassified PH was excluded. Ns include positives only (negatives constant at N = 161).

## Data Availability

Relevant de-identified subsets of the dataset may be shared with academic investigators on a case-by-case basis.

## Supplementary Materials

The following supporting information can be downloaded at: https://www.mdpi.com/article/10.3390/diagnostics14090897/s1, Supplement Section S1—Study Inclusion/Exclusion Criteria: Table S1: Inclusion Exclusion Criteria of IDENTIFY & CADLAD. Table S2: Inclusion Exclusion Criteria of IDENTIFY-PH. Table S3: Inclusion Exclusion Criteria of RADPH. Supplement Section S2—Features: Table S4: Analysis methods with description and utility of the model features [13,30,31,32,33,34,35,36,37,38,39,40,41,42,43,44,45]. Figure S1: Example of a feature capturing conduction pathway, as ventricular depolarization relates to repolarization, in a PH- subject (a), which exhibits a repolarization vector lying within the depolarization in phase as compared to a PH+ subject (b), in which repolarization is not enveloped by depolarization. Figure S2: Assessment of repolarization using wavelet time-frequency analysis, where, in contrast to PH- subjects (a), PH+ subjects tend to exhibit larger values of this feature, corresponding to longer repolarization, which is a characteristic indicative of repolarization deficits. Figure S3: Example of a PH- subject exhibiting dynamic changes in the respiration amplitude and frequency (a), PH+ subject exhibiting invariant respiration barring a single breath. Figure S4: Example of PH- subject (a) showing negative arterial compliance feature value (with visually identifiably low amplitude acceleration plethysmogram) and example of PH+ subject (b) showing positive arterial compliance feature value (with visually identifiably high amplitude acceleration plethysmogram). Figure S5: Example of a perfusion response to cardiac contraction feature, which examines the mutual information between the OVG and PPG signals, showing a high value in PH- (a), and lower value in PH+ (b). Figure S6: Example of atrial structure feature examining for the presence of additional deflections of notching in the atrial depolarization waveform, which are not present in the PH- subject (a), but are visible in the PH+ subject (b). Figure S7: Example of a feature capturing perfusion, as the red plethysmographic (blue trace) signals relates to infrared (red trace), demonstrating typical perfusion in a PH- subject (a) in contrast reduced perfusion in a PH+ subject (b).

## References

[1] Galié N., Humbert M., Vachiéry J.-L., Gibbs S., Lang I., Torbicki A., Simonneau G., Peacock A., Noordegraaf A.V., Beghetti M. (2015). 2015 ESC/ERS Guidelines for the diagnosis and treatment of pulmonary hypertension. Eur. Respir. J..

[2] Humbert M., Kovacs G., Hoeper M.M., Badagliacca R., Berger R.M., Brida M., Carlsen J., Coats A.J.S., Escribano-Subias P., Ferrari P. (2022). 2022 ESC/ERS Guidelines for the diagnosis and treatment of pulmonary hypertension: Developed by the task force for the diagnosis and treatment of pulmonary hypertension of the European Society of Cardiology (ESC) and the European Respiratory Society (ERS). Eur. Heart J..

[3] Dunlap B., Weyer G. (2016). Pulmonary Hypertension: Diagnosis and Treatment. Am. Acad. Fam. Physicians.

[4] Guazzi M., Borlaug B.A. (2012). Pulmonary hypertension due to left heart disease. Circulation.

[5] Vachiéry J.-L., Adir Y., Barberà J.A., Champion H., Coghlan J.G., Cottin V., De Marco T., Galiè N., Ghio S., Gibbs J.S.R. (2013). Pulmonary hypertension due to left heart diseases. J. Am. Coll. Cardiol..

[6] Lam C.S., Borlaug B.A., Kane G.C., Enders F.T., Rodeheffer R.J., Redfield M.M. (2009). Age-associated increases in pulmonary artery systolic pressure in the general population. Circulation.

[7] Galiè N., McLaughlin V.V., Rubin L.J., Simonneau G. (2019). An overview of the 6th World Symposium on Pulmonary Hypertension. Eur. Respir. J..

[8] Hoeper M.M., Humbert M., Souza R., Idrees M., Kawut S.M., Sliwa-Hahnle K., Jing Z.-C., Gibbs J.S.R. (2016). A global view of pulmonary hypertension. Lancet Respir. Med..

[9] Sikirica M., Iorga S.R., Bancroft T., Pot-ash J. (2014). The economic burden of pulmonary arterial hypertension (PAH) in the US on payers and patients. BMC Health Serv. Res..

[10] Janda S., Shahidi N., Gin K., Swiston J. (2011). Diagnostic accuracy of echocardiography for pulmonary hypertension: A systematic review and meta-analysis. Heart.

[11] Ong M., Klann J.G., Lin K.J., Maron B.A., Murphy S.N., Natter M.D., Mandl K.D. (2020). Claims-based algorithms for identifying patients with pulmonary hypertension: A comparison of decision rules and machine-learning approaches. J. Am. Heart Assoc..

[12] Burton T., Fathieh F., Nemati N., Gillins H.R., Shadforth I.P., Ramchandani S., Bridges C.R. (2024). Development of a Non-Invasive Machine-Learned Point-of-Care Rule-Out Test for Coronary Artery Disease. Diagnostics.

[13] Fathieh F., Paak M., Khosousi A., Burton T., Sanders W.E., Doomra A., Lange E., Khedraki R., Bhavnani S., Ramchandani S. (2021). Predicting cardiac disease from interactions of simultaneously-acquired hemodynamic and cardiac signals. Comput. Methods Programs Biomed..

[14] Nagueh S.F., Smiseth O.A., Appleton C.P., Byrd B.F., Dokainish H., Edvardsen T., Flachskampf F.A., Gillebert T.C., Klein A.L., Lancellotti P. (2016). Recommendations for the evaluation of left ventricular diastolic function by echocardiography: An update from the American Society of Echocardiography and the European Association of Cardiovascular Imaging. Eur. J. Echocardiogr..

[15] Bhavnani S.P., Khedraki R., Cohoon T.J., Meine F.J., Stuckey T.D., McMinn T., Depta J.P., Bennett B., McGarry T., Carroll W. (2022). Multicenter validation of a machine learning phase space electro-mechanical pulse wave analysis to predict elevated left ventricular end diastolic pressure at the point-of-care. PLoS ONE.

[16] Nemati N., Fathieh F., Burton T., Gillins H., Shadforth I., Ramchandani S., Bridges C.R. (2024). Development of a Non-Ivasive Point-of-Care Rule-Out Test for Hearth Failure Using Machine Learning. J. Am. Coll. Cardiol..

[17] Jia W., Sun M., Lian J., Hou S. (2022). Feature dimensionality reduction: A review. Complex Intell. Syst..

[18] Krittanawong C., Virk H.U.H., Bangalore S., Wang Z., Johnson K.W., Pinotti R., Zhang H., Kaplin S., Narasimhan B., Kitai T. (2020). Machine learning prediction in cardiovascular diseases: A meta-analysis. Sci. Rep..

[19] Breiman L. (2001). Random Forests. Mach. Learn..

[20] Zou H., Hastie T. (2005). Regularization and variable selection via the elastic net. J. R. Stat. Soc. Ser. B Stat. Methodol..

[21] Berrar D., Ranganathan S., Gribskov M., Nakai K., Schönbach C. (2019). Encyclopedia of Bioinformatics and Computational Biology.

[22] Breiman L. (2019). Bagging Predictors.

[23] Allgaier J., Mulansky L., Draelos R.L., Pryss R. (2023). How does the model make predictions? A systematic literature review on the explainability power of machine learning in healthcare. Artif. Intell. Med..

[24] Zehir R., Karabay C.Y., Kalaycı A., Akgün T., Kılıçgedik A., Kırma C. (2015). Evaluation of Tpe interval and Tpe/QT ratio in patients with slow coronary flow. Anatol. J. Cardiol..

[25] Moraes J.L., Rocha M.X., Vasconcelos G.G., Vasconcelos Filho J.E., De Albuquerque V.H.C., Alexandria A.R. (2018). Advances in photopletysmography signal analysis for biomedical applications. Sensors.

[26] Platonov P.G. (2012). P-wave morphology: Underlying mechanisms and clinical implications. Ann. Noninvasive Electrocardiol..

[27] Elgendi M. (2012). On the analysis of fingertip photoplethysmogram signals. Curr. Cardiol. Rev..

[28] Hoeper M.M., Badesch D.B., Ghofrani H.A., Gibbs J.S.R., Gomberg-Maitland M., McLaughlin V.V., Preston I.R., Souza R., Waxman A.B., Grünig E. (2023). Phase 3 trial of sotatercept for treatment of pulmonary arterial hypertension. N. Engl. J. Med..

[29] Heidenreich P.A., Bozkurt B., Aguilar D., Allen L.A., Byun J.J., Colvin M.M., Deswal A., Drazner M.H., Dunlay S.M., Evers L.R. (2022). 2022 AHA/ACC/HFSA guideline for the management of heart failure: Executive summary: A report of the American College of Cardiology/American Heart Association Joint Committee on Clinical Practice Guidelines. J. Am. Coll. Cardiol..

[30] Ayano Y.M., Schwenker F., Dufera B.D., Debelee T.G. (2023). Interpretable Machine Learning Techniques in ECG-Based Heart Disease Classification: A Systematic Review. Diagnostics.

[31] Allen J. (2007). Photoplethysmography and its application in clinical physiological measurement. Physiol. Meas..

[32] Pan D., Liu R., Ren S., Li C., Chang Q. (2016). Prediction of Pulmonary Arterial Hypertension in Chronic Obstructive Lung Disease from Three-Dimensional Vectorcardiographic Parameters. Ann. Noninvasive Electrocardiol..

[33] Rubulis A., Jensen J., Lundahl G., Tapanainen J., Wecke L., Bergfeldt L. (2004). T vector and loop characteristics in coronary artery disease and during acute ischemia. Heart Rhythm..

[34] Tereshchenko L.G., Waks J.W., Kabir M., Ghafoori E., Shvilkin A., Josephson M.E. (2015). Analysis of speed, curvature, planarity and frequency characteristics of heart vector movement to evaluate the electrophysiological substrate associated with ventricular tachycardia. Comput. Biol. Med..

[35] Sedaghat G., Ghafoori E., Waks J.W., Kabir M.M., Shvilkin A., Josephson M.E., Tereshchenko L.G. (2016). Quantitative assessment of vectorcardiographic loop morphology. J. Electrocardiol..

[36] Bansal D., Khan M., Salhan A.K. A Review of Measurement and Analysis of Heart Rate Variability. Proceedings of the 2009 International Conference on Computer and Automation Engineering.

[37] Călburean P.A., Pannone L., Sorgente A., Gauthey A., Monaco C., Strazdas A., Almorad A., Bisignani A., Bala G., Ramak R. (2023). Heart rate variability and microvolt T wave alternans changes during ajmaline test may predict prognosis in Brugada syndrome. Clin. Auton. Res..

[38] Wang T., Lu C., Sun Y., Yang M., Liu C., Ou C. (2021). Automatic ECG Classification Using Continuous Wavelet Transform and Convolutional Neural Network. Entropy.

[39] He R., Wang K., Zhao N., Liu Y., Yuan Y., Li Q., Zhang H. (2018). Automatic detection of atrial fibrillation based on continuous wavelet transform and 2D convolutional neural networks. Front. Physiol..

[40] Lin C.H. (2008). Frequency-domain features for ECG beat discrimination using grey relational analysisbased classifier. Comput. Math. Appl..

[41] Madsen H.M. (2017). Spectral Decomposition of Electrocardiograms for the Diagnosis of Pulmonary Hypertension and the Estimation of Invasively Measured Parameters. Master’s Thesis.

[42] Bhoi A.K., Sherpa K.S., Khandelwal A.B. (2018). Ischemia and arrhythmia classification using timefrequency domain features of QRS complex. Procedia Comput. Sci..

[43] PCharlton H., Bonnici T., Tarassenko L., Clifton D.A., Beale R., Watkinson P.J. (2016). An assessment of algorithms to estimate respiratory rate from the electrocardiogram and photoplethysmogram. Physiol. Meas..

[44] Schumann A., Wessel N., Schirdewan A., Osterziel K.J., Voss A. (2002). Potential of feature selection methods in heart rate variability analysis for the classification of different cardiovascular diseases. Stat. Med..

[45] Witte C., Meyer Zur Heide Genannt Meyer-Arend J.U., Andrié R., Schrickel J.W., Hammerstingl C., Schwab J.O., Nickenig G., Skowasch D., Pizarro C., Pokorski M. (2016). Heart Rate Variability and Arrhythmic Burden in Pulmonary Hypertension. Pulmonary Dysfunction and Disease. Advances in Experimental Medicine and Biology.

