# Supplementary material for: Pulmonary Hypertension Detection Non-Invasively at Point-of-Care Using a Machine-Learned Algorithm
Source: Diagnostics (Basel). 2024 Apr 25;14(9):897. doi: 10.3390/diagnostics14090897 (PMC11083349; doi:10.3390/diagnostics14090897)
Supplement: Supplementary file 1 [file diagnostics-14-00897-s001.zip › diagnostics-2929811-supplementary.pdf]

Supplement Section S1 – Study Inclusion / Exclusion Criteria

Table S1 – Inclusion Exclusion Criteria of IDENTIFY & CADLAD

Inclusion	Exclusion
1. ≥ 18 years of age<br>2. Cardiovascular symptoms<br>3. Scheduled to undergo cardiac catheterization with coronary angiography (Group 2 & CADLAD), SPECT MPI (Group 3) or CTA (Group 4)<br>4. Ability to understand the requirements of the study and to provide written informed consent	1. Prior documented history of myocardial infarction (MI)<br>2. Suspected acute myocardial infarction (AMI) at current presentation<br>3. Prior coronary artery bypass grafting (CABG)<br>4. Prior heart valve replacement<br>5. Previous sustained or paroxysmal atrial or ventricular arrythmia<br>6. Infiltrative myocardial disease (amyloid, sarcoid, right ventricular dysplasia)<br>7. Presence of cardiac implantable electronic device (CIED), including implantable cardioverter defibrillator (ICD), pacemaker (PM), implantable loop recorders and other monitors<br>8. Implantable Neuro-stimulators<br>9. Congenital Heart Disease<br>10. Pregnant or breast feeding<br>11. Currently taking any Type IA, IC or III antiarrhythmics<br>12. Any history of amiodarone use<br>13. Clinically significant chest deformity (e.g., pectus excavatum or pectus carinatum)<br>14. Breast implants<br>15. Neuromuscular disease if the condition results in tremor or muscle fasciculations

Table S2 – Inclusion Exclusion Criteria of IDENTIFY-PH

Inclusion	Exclusion
1. Patients≥ 18 years old.<br>2. Scheduled to undergo right heart catheterization.<br>3. Ability to understand the requirements of the study and to provide written informed consent.<br>4. Normal Sinus Rhythm (NS) at time of phase signal collection (study procedure).	5. Prior heart valve replacement or repair.<br>6. Prior lung or heart transplant.<br>7. Infiltrative myocardial disease (amyloid, sarcoid, right ventricular dysplasia).<br>8. Presence of cardiac implantable electronic device (CIED), including implantable cardioverter defibrillator (ICD), pacemaker (PM), implantable loop recorders and other monitors.<br>9. Implantable Neuro-stimulators.<br>10. Congenital Heart Disease.<br>11. Pregnant or breast feeding.<br>12. Currently taking any Type IA, IC or Ill antiarrhythmics.<br>13. Any history of amiodarone use.<br>14. Clinically significant chest deformity (e.g., pectus excavatum or pectus carinatum).<br>15. Breast implants.<br>16. Neuromuscular disease if the condition results in tremor or muscle fasciculations.

Table S3 – Inclusion Exclusion Criteria of RADPH

Inclusion	Exclusion
1. Patients ≥ 21 years old;<br>2. Previously undergone right heart catheterization (RHC), within 18 months of enrollment in study, showing recorded mAP >30 mm Hg; Sinus rhythm at the time of phase space signal recording<br>3. Ability to understand the requirements of the study and to provide written informed consent.	16. Prior hearth and/or transplant;<br>17. Prior heart valve replacement;<br>18. Presence of cardiac implantable electronic device<br>19. (CIED), including implantable cardioverter defibrillator<br>20. (ICD), pacemaker (PM), implantable loop recorders and other monitors;<br>21. Implantable Neuro-stimulators;<br>22. Congenital Heart Disease;<br>23. Pregnancy or Breast Feeding;<br>24. Clinically significant chest deformity (e.g., pectus excavatum or pectus carinatum);<br>25. Breast implants;<br>26. Neuromuscular Disease if the condition results in tremor or muscle fasciculations.

Supplement Section S2 – Features

Table S4. Analysis methods with description and utility of the model features.

Time Domain	
Description	Features that quantify OVG, PPG, the velocity plethysmogram (VPG), and the acceleration plethysmogram (APG) waveform attributes in the time domain.
Calculation	VPG and APG waveforms were generated by taking the first and second derivatives of the PPG signal. Cardiac cycles, peaks, onset and offset of the waveforms on OVG and PPG were detected using a discrete wavelet delineator (DWD). The detected fiducial points were used for feature calculation.
Sample Features	OVG features: P-wave, QRS, T-wave amplitudes, durations, R-R interval, heart rate, etc.<br>PPG-VPG-APG features: peak amplitude, slopes, presence/absence of dicrotic notch, systolic and diastolic area, slope ratios, AC to DC ratios etc. extracted from Red & IR waveforms.
Reported Utility	ECG feature: for the detection of several cardiac diseases such as cardiac ischemia, ventricular hypertrophy, arrythmia, PVC, atrial fibrillation, myocardial infarction, etc.[1]. PPG, VPG, and APG morphologies can be used to assess arterial compliance, cardiac sicknesses, and respiratory disorders[2].
Phase Space	
Description	Features that quantify the three-dimensional morphology of OVG and PPG signals in phase space.
Calculation	OVG phase space, commonly known as vectorcardiogram (VCG), was constructed using OVG three channels. Depolarization and repolarization waves were delineated using time domain DWD. PPG phase space was constructed using PPG, VPG, and APG signals. Features were extracted to measures 3D and 2D geometrical attributes for each cardiac loop and PPG cycles.
Sample Features	OVG: magnitude of P-QRS-T vectors, the direction of P-QRS-T vectors, P-QRS-T loop perimeter, P-QRS-T loop area, etc.<br>PPG: magnitude and direction of 3D and 2D maximal vectors, perimeter and volume of the 3D and 2D loops, etc.
Reported Utility	Detection of pulmonary arterial hypertension in chronic obstructive lung disease [3], and coronary artery diseases [4].
Wave Propagation	
Description	Features that quantify the propagative characteristics of the depolarization-repolarization waves in three-dimensional space for OVG.
Calculation	OVG phase space was constructed for P, QRS, and T loops; cardiac waves were delineated applying DWD to the signals in time domain. The propagation of depolarization and repolarization waves on time and space is performed on the bandpass. In addition, bandpass filters were applied to the input signal in the time domain and bandpass phase space was constructed. Residue phase space was constructed (the 3D point-cloud representing the Euclidian distance between the datapoints in the original phase space and the bandpass phase space). Bandpass phase space and residue phase space were used to extract 2D and 3D geometrical features.
Sample Features	Speed (a measure of cardiac loop propagation with time), curvature (a measure of cardiac loop deviation from a straight line), planarity (a measure of the mean of the dihedral angles between two consecutive planes for all planes generated for the median beat), and orbital frequency (a scalar measure of rotation rate of the cardiac vector), residue cloud volume, surface area, porosity, density, etc.
Reported Utility	Detection of sustained monomorphic ventricular tachycardia (VT)[5] and diagnosis of acute myocardial infarction[6].
Dynamics and Variabilities	
Description	Features that quantify dynamical behaviour of the OVG and PPG and their variabilities with time.

---

1 Ayano, Y.M.; Schwenker, F.; Dufera, B.D.; Debelee, T.G. Interpretable Machine Learning Techniques in ECG-Based Heart Disease Classification: A Systematic Review. *Diagnostics* **2023**, *13*, 111. https://doi.org/10.3390/diagnostics13010111

2 Allen, John. "Photoplethysmography and its application in clinical physiological measurement." *Physiological measurement* 28.3 (2007): R1.

3 Pan, D., Liu, R., Ren, S., Li, C. and Chang, Q. (2016), Prediction of Pulmonary Arterial Hypertension in Chronic Obstructive Lung Disease from Three-Dimensional Vectorcardiographic Parameters. Annals of Noninvasive Electrocardiology, 21: 280-286.

4 Rubulis, A., Jensen, J., Lundahl, G., Tapanainen, J., Wecke, L., & Bergfeldt, L. (2004). T vector and loop characteristics in coronary artery disease and during acute ischemia. Heart rhythm, 1(3), 317–325.

5 Tereshchenko LG, Waks JW, Kabir M, Ghafoori E, Shvilkin A, Josephson ME. Analysis of speed, curvature, planarity and frequency characteristics of heart vector movement to evaluate the electrophysiological substrate associated with ventricular tachycardia. Comput Biol Med. 2015 Oct 1;65:150-60.

6 Sedaghat, Golriz, et al. "Quantitative assessment of vectorcardiographic loop morphology." Journal of electrocardiology 49.2 (2016): 154-163.

Calculation	Dynamical analysis was performed using linear and non-linear techniques on OVG and PPG. Poincare 2D maps were construed to capture the system's behaviour at specific state space. In addition, beat-to-beat variabilities in the OVG were computed by comparing each beat to the template beat, the most prominent waveform represented across the entire signal. The residue waveform was constructed by finding the Euclidian distance between the segmented beats and the template beat. Distribution of the residue was analyzed to compute variability features.
Sample Features	Lyapunov exponent, correlation, entropy, mutual information, correlation, Poincare circularity, Poincare spread ratio, residue mean, residue standard deviation, residue skewness, etc.
Reported Utility	Several studies reported that heart rate variability and wave pattern changes have predictive power for the detection of cardiac diseases[7,8].
Time-Frequency	
Description	Features that quantity the frequency content of a signal over cardiac waves period and PPG cycles.
Calculation	The cardiac wave (P, QRS, T) and PPG cycles were segmented using DWD and then transformed from the time domain to the time-frequency domain using Continuous Wavelet Transform (CWT). The CWT scalograms were divided into high and low power regions using image processing and statistical techniques, where image-based features were extracted from those regions.
Sample Features	Time centroid, frequency centroid, maximum energy, time span, frequency span, number of regions, etc.
Reported Utility	Detection of several cardiac diseases, such as abnormal cardiac muscle relaxation, coronary artery disease, ventricular fibrillation, and atrial fibrillation[9,10].
Frequency Spectrum	
Description	Features that quantify the power spectrum and frequency content of the PPG and OVG.
Calculation	Fast-Fourier Transform (FFT) was applied to each channel of OVG and PPG (Red-IR) to obtain a power spectrogram.
Sample Features	Fundamental frequency, harmonic amplitude, harmonic decay rate, spectral statistical distribution measures (e.g. std, skewness).
Reported Utility	Classification of heart-beat classes (i.e. normal beats, supraventricular ectopic beat, bundle branch ectopic beat, and cardiac arrhythmias)[11], detection of pulmonary hypertension[12] and ischemia[13].
Respiration	
Description	Features that quantify the characterises of the approximated respiration waveform using both the PPG (i.e. Red and IR) and OVG signals (three channels).
Calculation	The respiration waveforms were estimated by capturing the amplitude modulation and frequency modulation in OVG and PPG signals following the techniques presented in literature[14]. Modulation signals and the respiration proxy were used for feature extraction.
Sample Features	Heart rate variability (HRV), respiration rate (mean, std, skewness), amplitude and frequency modulation (mean, std, skewness, etc.)

[7] D. Bansal, M. Khan, and A. K. Salhan, "A Review of Measurement and Analysis of Heart Rate Variability," in 2009 International Conference on Computer and Automation Engineering, 2009, pp. 243–246.

[8] P. A. Călburean et al., "Heart rate variability and microvolt T wave alternans changes during ajmaline test may predict prognosis in Brugada syndrome," Clin. Auton. Res., vol. 33, no. 1, pp. 51–62, Feb. 2023.

[9] Wang, T.; Lu, C.; Sun, Y.; Yang, M.; Liu, C.; Ou, C. Automatic ECG Classification Using Continuous Wavelet Transform and Convolutional Neural Network. Entropy 2021, 23, 119.

[10] He, R., Wang, K., Zhao, N., Liu, Y., Yuan, Y., Li, Q., & Zhang, H. (2018). Automatic detection of atrial fibrillation based on continuous wavelet transform and 2D convolutional neural networks. *Frontiers in physiology*, *9*, 1206.

[11] C. H. Lin, "Frequency-domain features for ECG beat discrimination using grey relational analysisbased classifier," comput. math. with appl., vol. 55, no. 4, pp. 680–690, 2008.

[12] H. M. Madsen, "spectral decomposition of electrocardiograms for the diagnosis of pulmonary hypertension and the estimation of invasively measured parameters," M.S. thesis, College of BioEng., Univ of Colorado, Colorado, 2017.

[13] A. K. Bhoi, K. S. Sherpa, And B. Khandelwal, "Ischemia and arrhythmia classification using timefrequency domain features of QRS complex," procedia computer science, vol. 132, no. 4, pp. 606613, 2018.

[14] P. H. Charlton, T. Bonnici, L. Tarassenko, D. A. Clifton, R. Beale, and P. J. Watkinson, "An assessment of algorithms to estimate respiratory rate from the electrocardiogram and photoplethysmogram," Physiol. Meas., vol. 37, no. 4, pp. 610–626, Mar. 2016.

Reported Utility	Characterize patients suffering from coronary heart disease (CHD), dilated cardiomyopathy (DCM) and patients who had survived an acute myocardial infarction (MI)[15]. Possible utility in the detection of pulmonary arterial hypertension[16].

Cardiac-PPG Interactions

Description	Features that quantify the synchronicity between the OVG and PPG.
Calculation	Features calculated using both components of the PPG signal (1st modality) as well as all three channels of the OVG signal (2nd modality) to characterize the synchronicity between the two modalities using Poincare maps[17].
Sample Features	Poincare distribution features such as mean, std, skewness and shape feature such as circularity, circumferences, etc. Pulse transit time using OVG and PPG.
Reported Utility	Separation of asymptomatic subjects from patients with CAD and Elevated Left Ventricular End Diastolic Pressure (LVEDP)[18]

[15] Schumann, Agnes, et al. "Potential of feature selection methods in heart rate variability analysis for the classification of different cardiovascular diseases." *Statistics in medicine* 21.15 (2002): 2225-2242.
[16] Witte, C. *et al.* (2016). Heart Rate Variability and Arrhythmic Burden in Pulmonary Hypertension. In: Pokorski, M. (eds) Pulmonary Dysfunction and Disease. Advances in Experimental Medicine and Biology(), vol 934. Springer, Cham.
[17] Fathieh, F., Paak, M., Khosousi, A., Burton, T., Sanders, W. E., Doomra, A., ... & Ramchandani, S. (2021). Predicting cardiac disease from interactions of simultaneously-acquired hemodynamic and cardiac signals. *Computer Methods and Programs in Biomedicine*, *202*, 105970.
[18] Fathieh, F., Paak, M., Khosousi, A., Burton, T., Sanders, W. E., Doomra, A., ... & Ramchandani, S. (2021). Predicting cardiac disease from interactions of simultaneously-acquired hemodynamic and cardiac signals. *Computer Methods and Programs in Biomedicine*, *202*, 105970.

Conduction Features: These features measure characteristics of myocardial conduction pathway and variations in that pathway using the OVG signal. For example, the relationship between the depolarization vector in the three-dimensional phase space formed by the three OVG channels shows significant differences between PH- and PH+ (examples in Figure S1).

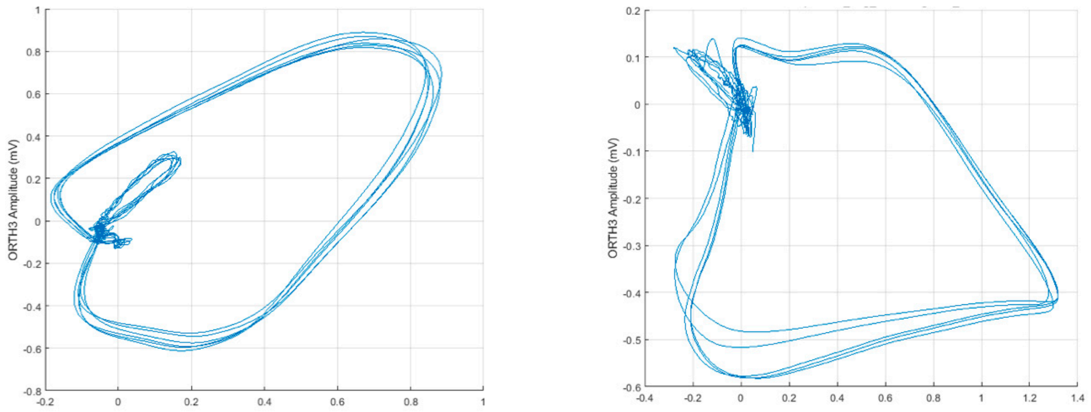

(a) PH- Example (b) PH+ Example

Figure S1: Example of a feature capturing conduction pathway, as ventricular depolarization relates to repolarization, in a PH- subject (a), which exhibits a repolarization vector lying within the depolarization in phase as compared to a PH+ subject (b), in which repolarization is not enveloped by depolarization.

Repolarization Features: These features measure the properties of myocardial repolarization. An example repolarization feature, shown is Figure S2, involves wavelet time-frequency analysis to detect the high-power region in the T-wave, and measurement of those duration of that region over the course of the signal. PH+ subjects tend to exhibit larger values of this feature, corresponding to longer repolarization, which is a characteristic indicative of repolarization deficits.

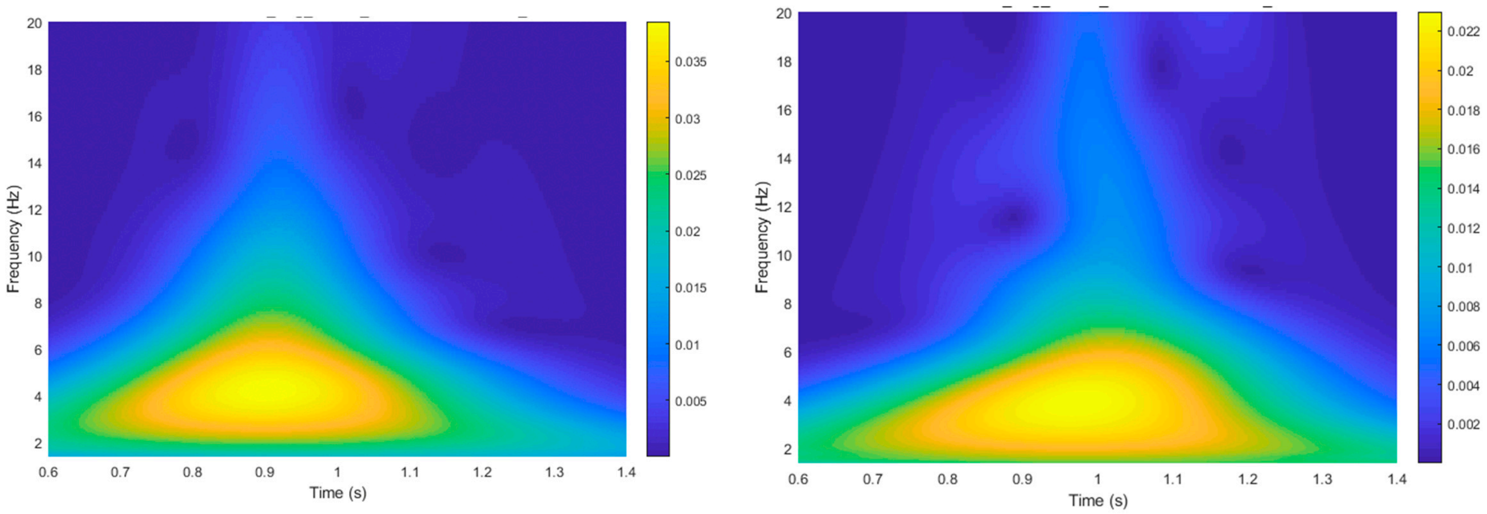

(a) PH- Example (a) (b) PH+ Example

Figure S2: Assessment of repolarization using wavelet time-frequency analysis, where, in contrast to PH- subjects (a), PH+ subjects tend to exhibit larger values of this feature, corresponding to longer repolarization, which is a characteristic indicative of repolarization deficits.

Respiration Features: These features estimate the respiration waveform and evaluate characteristics of that estimation, using the PPG and OVG signals. PH- subjects tend to have more dynamic estimated respiration, perhaps similar to heart rate variability in ability to adapt to changes intrinsic and extrinsic conditions. In contrast, PH+ subjects tend to have less dynamic, more invariant estimated respiration, with lowered adaptation ability (Figure S3).

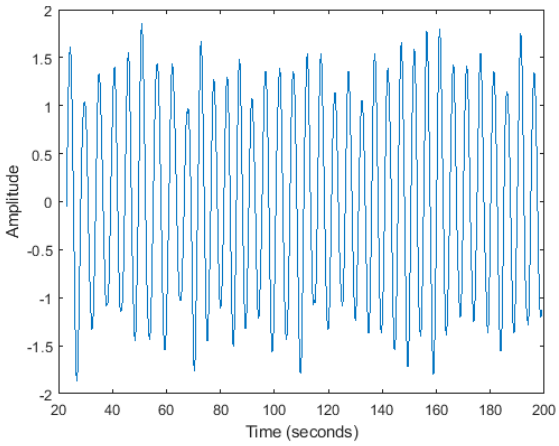

(a) PH- Example

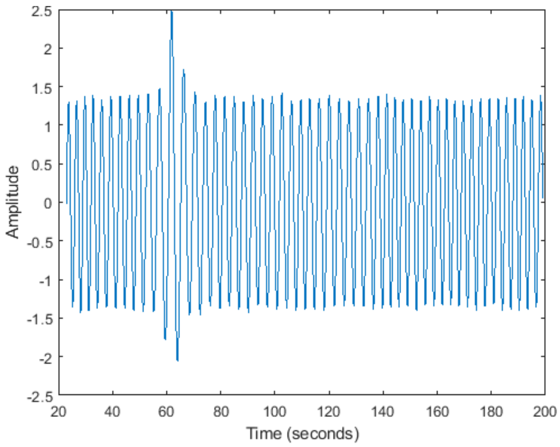

(b) PH+ Example

Figure S3: Example of a PH- subject exhibiting dynamic changes in the respiration amplitude and frequency (a), PH+ subject exhibiting invariant respiration barring a single breath.

Arterial Compliance Features: This category of features captures arterial compliance (i.e., increasing stiffness) using combinations of plethysmogram itself, the velocity plethysmogram (1st derivative) and acceleration plethysmogram (2nd derivative). An example of the plethysmogram and acceleration plethysmogram are shown in Figure S4, from which an angle feature is calculated in phase space.

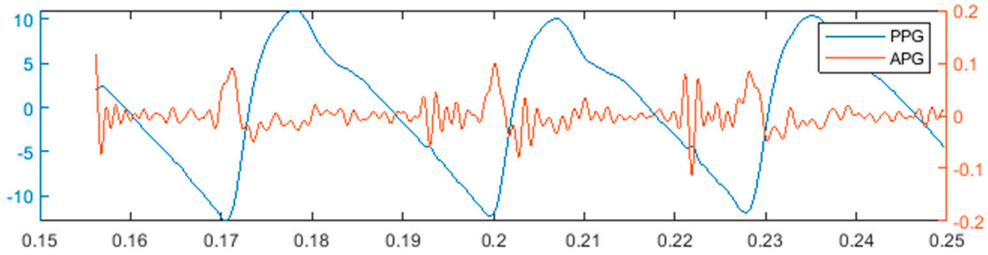

(a) PH- Example

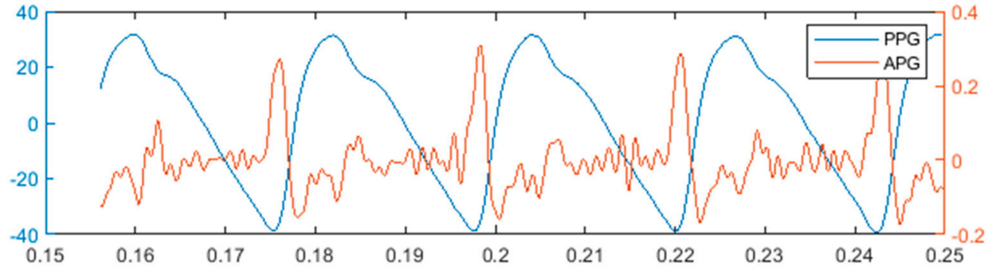

(b) PH+ Example

Figure S4: Example of PH- subject (a) showing negative arterial compliance feature value (with visually identifiably low amplitude acceleration plethysmogram) and example of PH+ subject (b) showing positive arterial compliance feature value (with visually identifiably high amplitude acceleration plethysmogram)

Perfusion Response to Cardiac Contraction: These features analyze the relationship of the PPG signal to the OVG signal – dynamics of the interplay between blood flow and myocardial conduction characteristics could indicate differential cardiac function, reflecting underlying pathophysiology. An example is shown in Figure S5, which examines the mutual information between the OVG and PPG signals, where PH- subjects tend to have more information shared between those modalities than PH+.

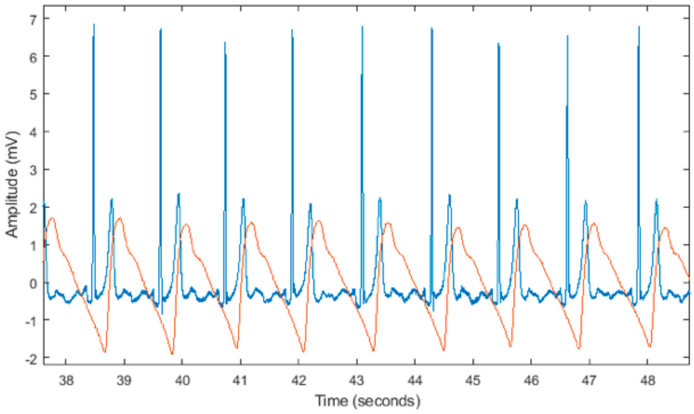

(a) PH- Example

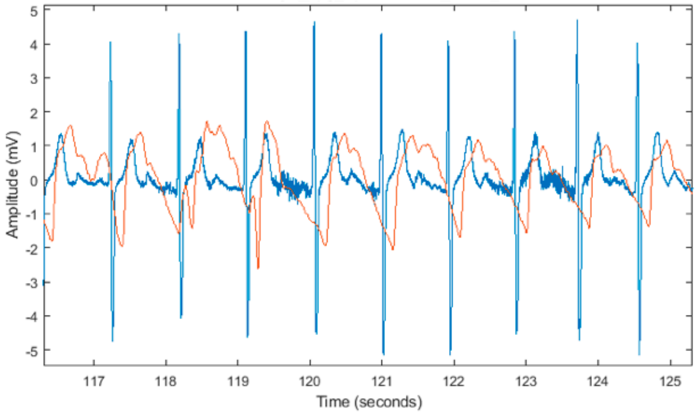

(b) PH+ Example

Figure S5: Example of a perfusion response to cardiac contraction feature, which examines the mutual information between the OVG and PPG signals, showing a high value in PH- (a), and lower value in PH+ (b).

Atrial Structure: These features capture elements of trial structure; for example, the complexity of atrial depolarization (e.g., presence of secondary waveforms), as shown in Figure S6.

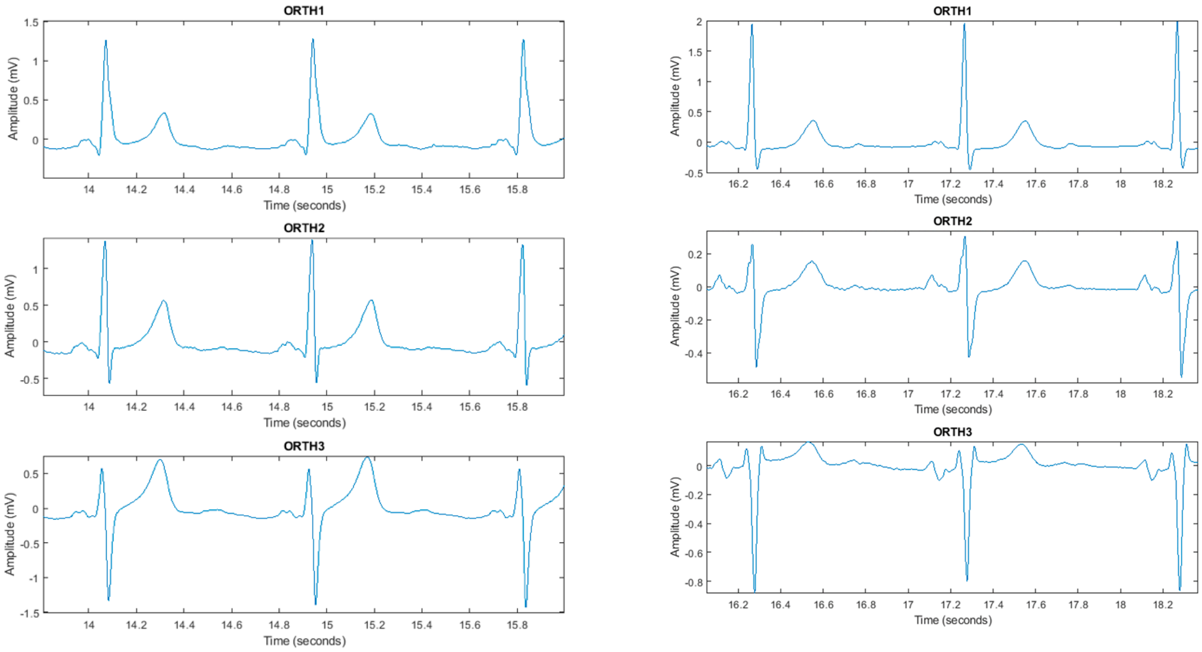

(a) PH- Example (b) PH+ Example

Figure S6: Example of atrial structure feature examining for the presence of additional deflections of notching in the atrial depolarization waveform, which are not present in the PH- subject (a), but are visible in the PH+ subject (b).

Perfusion Features: These features capture the shape of the PPG waveform itself, as well as the relationship between the infrared and red PPG signals. For example, comparison of the AC and DC amplitudes of red and infrared, which indicates that PH+ subjects tend to have reduced perfusion as compared to PH- (examples in Figure S7).

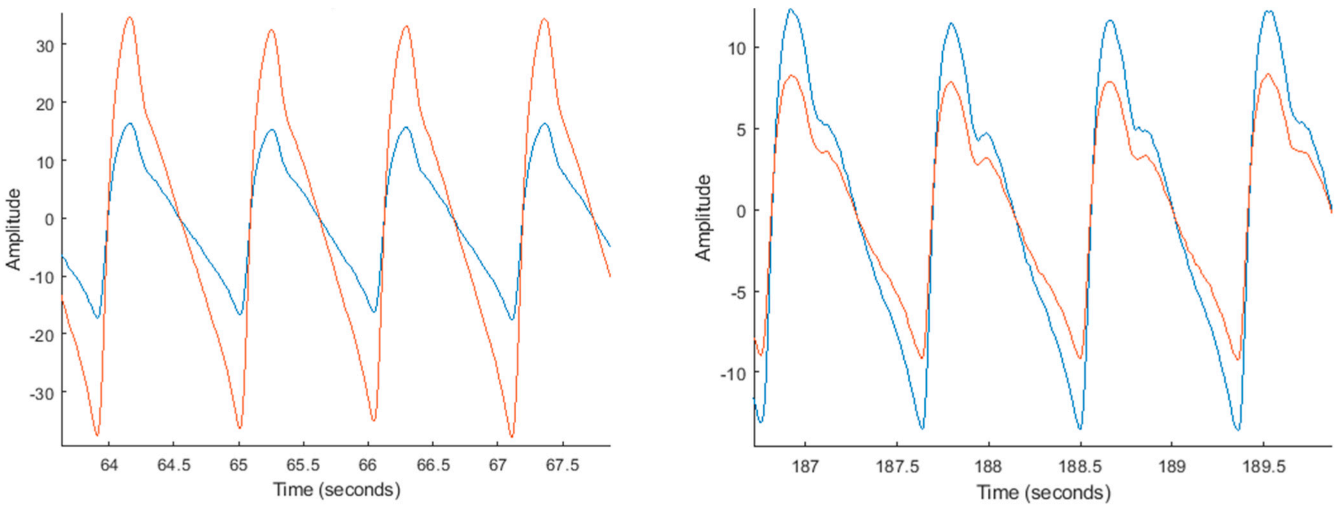

(a) PH- Example

(b) PH+ Example

Figure S7: Example of a feature capturing perfusion, as the red plethysmographic (blue trace) signals relates to infrared (red trace), demonstrating typical perfusion in a PH- subject (a) in contrast reduced perfusion in a PH+ subject (b).
